# The “Glymphatic-Lymphatic System Pathology” and a New Categorization of Neurodegenerative Disorders

**DOI:** 10.3389/fnins.2021.669681

**Published:** 2021-05-20

**Authors:** Pasquale Gallina, Claudio Nicoletti, Antonio Scollato, Francesco Lolli

**Affiliations:** ^1^Department of Neurosciences, Psychology, Drug Research and Child Health, University of Florence, Florence, Italy; ^2^Neurosurgical Unit, Careggi University Hospital, Florence, Italy; ^3^Department of Experimental and Clinical Medicine, Section of Anatomy, University of Florence, Florence, Italy; ^4^Neurosurgical Unit, Cardinale Panico Hospital, Lecce, Italy; ^5^Department of Clinical and Experimental Biomedical Sciences “Mario Serio”, University of Florence, Florence, Italy

**Keywords:** neurodegeneration, neurodegenerative disorders, CSF dynamic disturbance, perivascular spaces of the brain, glymphatic, glymphatic circulation, lymphatic vessel

## Introduction

Neurodegeneration is the ultimate manifestation of various neurodegenerative, neurovascular, and neuroinflammatory diseases, brain traumatisms, and aging. However, viewing neurodegeneration as the pathological/clinical epiphenomenon of numerous specific pathogenetic mechanisms has not provided a unitary neurodegenerative model nor a cure.

It is emerging that neurodegeneration is related to failure of the glymphatic-lymphatic system(G-Ls) (Nedergaard and Goldman, 2020). This system represents a functionally integrated unit regulating solute trafficking and immune-surveillance in the CNS (Louveau et al., 2018; Nedergaard and Goldman, 2020). The G-Ls sub-serves CSF's flow from the subarachnoid spaces into the perivascular spaces and subsequently into the interstitium, and the aquaporin-4 water channels drive it. The cerebrospinal-interstitial fluid then passes to the venous perivascular and perineural spaces, lastly draining toxic molecules and immune cells from the brain into meninges and deep lymph nodes.

If we think of G-Ls dysfunction as the common final pathway to neurodegeneration (Nedergaard and Goldman, 2020), we can no longer consider the heterogeneous clinical cases where neurodegeneration manifests distinctively as diseases *per se*, but pathologies of G-Ls where *noxae* and symptoms are centered on a unique physiopathology. We propose a systematic vision of the events leading to the “G-Ls pathology” and, according to the relevant G-Ls dysfunctions, introduce a new categorization of diseases manifesting with neurodegeneration.

## Opinion

Primary or secondary *noxae* may affect the G-Ls. Primary *noxae* are the genetic aquaporin-4 channelopathies (Rainey-Smith et al., 2018) or auto-antibodies that directly damage aquaporin-4 (Papadopoulos and Verkman, 2013). Secondary *noxae* are the derangements of the intra-extra cranial hydrodynamic at various levels leading to CSF circulation alterations (Wilson, 2016; Tuovinen et al., 2020; Rajna et al., 2021). CSF flow waves, hemodynamic, and neuronal electrical activity oscillations connect in sleep (Fultz et al., 2019). CSF flow failure follows a reduction of arterial pulsatility (Mestre et al., 2018; Daversin-Catty et al., 2020) and an increase in intracranial venous pressure due to the restriction of intracranial veins or increased extracranial venous pressure transmitted to the brain (Wilson, 2016). CSF flow suppress during breath-holding (Dreha-Kulaczewski et al., 2015, 2017). Moreover, debris in the CSF spaces or their obstruction for congenital, inflammatory, infectious, haemorrhagic, neoplastic, and traumatic processes impairs CSF circulation, see Table 2 in Williams et al. (2019).

Failure of the CSF dynamic interacts with G-Ls flow processes and triggers the “G-Ls pathology.“ If CSF does not flow efficiently, it stagnates and accumulates in the perivascular spaces, expanding and becoming visible at imaging, as observed in neurodegeneration conditions (Wardlaw et al., 2020). Also, minor changes in CSF pressure, tissue pressure, or vascular function may alter the shape of perivascular spaces, leading to an altered flow dynamic in perivascular spaces and secondary flow impairment (Wardlaw et al., 2020). Arteriolar hypopulsatility also hampers perivascular CSF outflow (Mestre et al., 2018), as hypothesized when global CSF circulation stops (Gallina et al., 2019a). Experimental evidence supports this mechanism, since decreasing plasma osmolality, functionally simulating perivascular spaces congestion, leads to slowed G-Ls inflow (Plog et al., 2018). Furthermore, impairment of respiratory motion might reduce perivascular spaces flushing (Wardlaw et al., 2020) and failure of the perivascular CSF outflow leads to ”perivascular spaces damage.“

Perivenular involvement may also occur (Wilson, 2016). Therefore, cerebrospinal-interstitial fluid might also stagnate in the interstitium due to the rise of resistance on the venous side of perivascular spaces from decreased fluid efflux (Jiang et al., 2017). The interstitial spaces are then engulfed, interstitial bulk flow blocked, and interstitial and likely intracranial pressures increase (Lenck et al., 2018). Disturbances at the interstitium level may, in addition, damage the perivascular astrocyte endfeet, with injury of aquaporin-4 water channels, a further slowing down of interstitial fluid flow, and self-sustained interstitial congestion (Simon and Iliff, 2016). The events at the level of interstitial spaces configure the ”interstitial damage.“ Neuroinflammation and reactive gliosis perpetuate the *noxa*e by reverberating the above-mentioned pathological steps in a vicious circle.

## Discussion

Considering how G-Ls functions (Louveau et al., 2018; Nedergaard and Goldman, 2020), its chronic failure may lead to dysregulation of brain waste molecule clearance, intra-extracellular water, ion homeostasis, and CNS immune response alterations. Moreover, since aquaporin-4 is involved in ependymal development and physiology (Kahle et al., 2016), G-Ls damage may disrupt the anatomy and function of CSF spaces.

We categorize disorders culminating in neurodegeneration with a new path: (1) proteinopathies; (2) interstitial/cellular water accumulation and neural hyperexcitation pathologies; (3) autoimmune brain pathologies; and (4) CSF resorption and flow disturbances.

In the “G-Ls pathology” model, therapies to prevent neurodegeneration should target aquaporin-4 polymorphisms and alterations; therapies aimed at preventing aquaporin-4-specific IgG binding are under investigation (Papadopoulos and Verkman, 2013). Moreover, the prevention of hydrodynamic disturbances related to arterial circulation can follow treatment indications for cerebrovascular disease (Smith et al., 2017). The efficiency of systemic arterial flow may avoid CSF stagnation at the perivascular spaces, while implantable pump methods can regulate the CSF pulsatility by volume changes in low blood flow situations in the canine (Luciano et al., 2017). On the venous side, the search and correction of possible causes of intracranial venous hypertension (Wilson, 2016), including hepatic (Gallina et al., 2019b; Hadjihambi et al., 2019) and pulmonary dysfunctions (Gallina et al., 2020; Kananen et al., 2020; Russ et al., 2020; Tuovinen et al., 2020), may prevent or restore a reduction of the blood brain flow and facilitate CSF circulation. Moreover, CSF drainage may reverse venous hypertension, restoring interstitial fluid circulation (Wilson, 2016). Yet, CSF cleaning by shunting can minimize the alteration of the anatomy of subarachnoid spaces at the time of the injury (Abolfazli et al., 2016). At the same time, endothelial growth factor treatments promise to correct meningeal lymphatic vessel dysfunctions (Da Mesquita et al., 2018). Overall, interventions at the intra-extracranial hydrodynamic system level may prevent CSF circulation failure and “perivascular space damage.” In parallel, intervening on the CSF circulation dynamic by shunting can target “interstitial damage,“ creating more room for the interstitial space (Graff-Radford, 2014). The quality of night's sleep guards neurotoxic proteins' clearance, and therapies targeting molecular mechanisms might promote G-Ls repair (Boland et al., 2018). Finally, since inflammation plays a role at all of the proposed cascade levels, anti-inflammatory treatment might be helpful in various steps of the process. It would be crucial to understand the time-course of the sequence of events in the “G-Ls pathology” and the threshold of possible reversibility in any of its steps to plot new therapeutic strategies. To this end, the possibility of using dynamic magnetic resonance imaging to track G-Ls, in small laboratory animals (Xue et al., 2020) and humans (Rajna et al., 2021), at various steps, might prove instrumental in achieving this goal.

## Conclusion

In our opinion, neurodegeneration is no longer the result of several heterogeneous brain diseases but the ultimate clinical/pathological expression of the unique ”G-Ls pathology," as the consequence of multiple factors classifiable in the frame of cerebral hydrodynamic disturbances.

## Author Contributions

PG and FL conceived the study, acquired the data, and wrote the paper. AS and CN revised the paper critically for important intellectual content. All authors gave final approval of the version to be published.

## Conflict of Interest

The authors declare that the research was conducted in the absence of any commercial or financial relationships that could be construed as a potential conflict of interest.
